# Long-term outcomes of colorectal endoscopic submucosal dissection in elderly patients

**DOI:** 10.1007/s00384-016-2719-y

**Published:** 2016-11-30

**Authors:** Yoshifumi Takahashi, Ken-ichi Mizuno, Kazuya Takahashi, Hiroki Sato, Satoru Hashimoto, Manabu Takeuchi, Masaaki Kobayashi, Junji Yokoyama, Yuichi Sato, Shuji Terai

**Affiliations:** 1 Graduate School of Medical and Dental Science, Department of Gastroenterology and Hepatology, Niigata University, 757-1, Asahimachidori, Chuo-ku, Niigata City, Niigata 951-8510 Japan; 2 Department of Gastroenterology and Hepatology, Nagaoka Red Cross Hospital, Nagaoka, Japan; 3 Department of Gastroenterology and Hepatology, Uonuma Institute of Community Medicine, Niigata University Medical and Dental Hospital, Niigata, Japan

**Keywords:** Colorectal cancer, Elderly, Endoscopic submucosal dissection

## Abstract

**Background and aims:**

The safety and efficacy of endoscopic submucosal dissection (ESD) in elderly patients remain unclear. The aim of this study is to clarify the short- and long-term outcomes of colorectal ESD in elderly patients.

**Patients and methods:**

A total of 482 consecutive patients with 501 colorectal lesions treated with ESD from February 2005 to December 2013 were retrospectively reviewed. Patients were divided into two groups: an elderly group (≥ 75 years of age) and a non-elderly group (< 75 years of age). Short-term outcomes of interest were procedure time, complication rate, hospital stay, en bloc resection rate, and non-curative resection rate. Long-term outcomes of interest were disease-specific survival, and overall survival rates in the elderly group (51 patients) and non-elderly group (92 patients) were also analyzed.

**Results:**

No significant differences were observed between the groups with respect to short-term outcomes. Two patients in each group required emergency surgery. Of the patients who underwent non-curative resection, 7/12 (58%) in the elderly group and 15/23 (65%) in the non-elderly group underwent additional surgery. The 5-year disease-specific survival rates in the elderly and non-elderly groups were both 100%, and the corresponding 5-year overall survival rates were 86.3 and 93.5%, respectively (*p* = 0.026).

**Conclusions:**

Short-term outcomes after colorectal ESD were equivalent in both groups, and all patients showed favorable long-term outcomes. Considering the benign prognosis of lesions resected with ESD, preoperative screening of comorbidities is essential to improve overall survival.

## Introduction

The aging of the global population is a worldwide problem and has a significant effect on clinical practice [1]. In Japan, there has also been an increase in the number of elderly citizens, and the proportion of people aged older than 75 years reached 12.9% in 2015 [2]. Elderly patients usually present with comorbidities; therefore, indications for surgical treatment based on age have traditionally been debated [3, 4].

Colorectal cancer (CRC) is one of the most common diseases in the elderly, and the incidence of CRC has been rapidly increasing in Asian and Western countries. While surgical resection is the main treatment for CRC, this method may not be appropriate or safe in elderly patients with comorbidities. Meanwhile, endoscopic resection has been developed as a minimally invasive procedure for early CRC and precancerous lesions. Endoscopic submucosal dissection (ESD) has become widespread because of its high en bloc resection rate. However, ESD is not without its complications, which include a risk of perforation and bleeding [5–8]. The increasing incidence of CRC has led to the increased use of ESD procedures in elderly patients. However, few reports have discussed ESD in the elderly [9, 10]. In the present study, we aimed to assess the safety and efficacy of ESD in elderly patients, and investigated age-specific differences in short- and long-term outcomes following ESD.

## Materials and methods

A total of 482 consecutive patients with 501 colorectal lesions treated with ESD at the Niigata University Medical and Dental Hospital from February 2005 to December 2013 were retrospectively reviewed. All lesions were observed via magnifying chromoendoscopy before the ESD procedure [11, 12]. The indications for colorectal ESD were as follows: estimated depth of submucosal invasion of less than 1000 μm, tumor size larger than 20 mm, or tumor less than 20 mm in diameter showing non-lifting signs.

The patients were divided into two groups: an elderly group consisting of patients ≥75 years of age and a non-elderly group consisting of patients <75 years of age. Tumor size, procedure time, complication rate (perforation and bleeding), hospital stay, en bloc resection rate, non-curative resection rate, and management of non-curative resection cases were compared between these two groups.

### ESD procedure

Bowel irrigation was performed with 2–3 L of polyethylene glycol solution before the procedure. ESD was performed using a single-channel gastrointestinal endoscope (Olympus PCF TYPE Q260AI or PCF TYPE Q260JI, Olympus Medical Systems, Co. Ltd., Tokyo, Japan) and a high-frequency generator with an automatically controlled system (ICC200 or VIO300D, Erbe Elektromedizin Ltd., Tűbingen, Germany). The solution for submucosal injection was a mixture of 10% glycerol and epinephrine. When long-lasting elevation of the submucosa was required, hyaluronic acid solution (Mucoup; Johnson & Johnson K.K., Tokyo, Japan) was injected [13, 14]. The DualKnife (KD-650Q, Olympus Medical Systems Co. Ltd.), HookKnife (KD-620QR, Olympus Medical Systems Co. Ltd.), and FlushKnife (DK2618JN20, Fujinon Co., Tokyo, Japan) insulated tip knives were used for ESD, and the Coagrasper (FD-411QR, Olympus Medical Systems Co. Ltd.) was used for coagulation and hemostasis. Intravenous midazolam or dexmedetomidine hydrochloride was administered during all ESD procedures. Carbon dioxide insufflation was used during all ESD procedures instead of air insufflation to reduce patient discomfort as well as gas leakage in the case of an intraoperative perforation.

### Histopathological assessment

All resected specimens were fixed in 10% buffered formalin and cut into 2-mm slices. Hematoxylin-eosin-stained sections were histopathologically examined by pathologists in our hospital. The criteria for curative resection were as follows: the lateral and vertical margins of the specimen were free of cancer, submucosal invasion was less than 1000 μm from the muscularis mucosae, there was no evidence of lymphatic or vascular invasion, and there were no poorly differentiated components. Histological diagnoses were based on the Japanese classification system for cancer of the colon and rectum and the Vienna classification system [15].

### Follow-up and collection of long-term outcome data

Follow-up surveillance endoscopy was performed 6–12 months after ESD. Annual surveillance endoscopies and contrasted-enhanced computed tomography (CT) scans were scheduled for patients with non-curative resections.

The long-term outcome data of patients who underwent colorectal ESD were collected retrospectively from January 2014 to March 2014. Patients who underwent colorectal ESD from February 2005 to December 2008 were enrolled in the analysis of long-term outcomes. For patients who had not visited our hospital regularly, the long-term outcome data were retrieved by request from the referring physicians, or through a mail questionnaire or structured telephone interview. Survival time was calculated as the interval between the date of the first treatment and the date of death or the last date on which the patient was confirmed to be alive. This study was approved by the ethics committee of the school of medicine, Niigata University, Japan. Written informed consent to participate was obtained from all patients who underwent colorectal ESD.

### Statistical analysis

All variables in this study were analyzed with the SPSS version 17 software (SPSS Japan Inc., Tokyo, Japan). The variables between the two groups were analyzed with independent Student’s *t* test or Mann–Whitney *U* test. A *χ*2 test and Fisher’s exact test were performed for categorical variables. Overall survival rates were calculated by using the Kaplan-Meier method with comparison between the groups using the log-rank test. All tests of significance were two-tailed, and *p* values <0.05 were considered statistically significant.

## Results

### Patient characteristics and lesions

A total of 482 consecutive patients with 501 colorectal lesions were treated with ESD. Of these, 157 were in the elderly group (mean age 79.3 years, range 75–90), and 325 were in the non-elderly group (mean age 63.9 years, range 27–74). Patient characteristics are summarized in Table 1. There were no significant differences between the two groups with respect to sex ratio, tumor location, tumor size, or morphology. A total of 11 lesions in the elderly group and 19 in the non-elderly group were diagnosed on histopathology as deep invasive submucosal cancer. There were no significant differences in the histopathological assessments.

**Table 1:** Patient and tumor characteristics

	Elderly patients	Non-elderly patients	*p* value
Patients (*n*)	157 (32.6%)	325 (67.4%)	
Age (years), mean (range)	79.3 (75–90)	63.9 (27–74)	
Sex, male/female	92/65	197/128	0.67
Lesion (*n*)	164 (32.7%)	337 (67.3%)	
Tumor location (*n*)			
Cecum and colon	112 (68.3%)	227 (67.4%)	0.83
Rectum	52 (31.7%)	131 (32.6%)	
Tumor size			
Long axis (mm), mean ± SD	37.0 ± 16.5	35.2 ± 15.9	0.26
Short axis (mm), mean ± SD	29.7 ± 15.4	27.7 ± 13.6	0.23
Morphology (*n*)			
LST-G	93 (56.7%)	173 (51.3%)	0.68
LST-NG	55 (33.5%)	122 (36.2%)	
Protruded	14 (8.5%)	34 (10.1%)	
Depressed	0 (0.0%)	2 (0.6%)	
Residual/local recurrence	2 (1.2%)	6 (1.8%)	
Histological classification (*n*)			
Adenoma	52 (31.7%)	131 (38.9%)	0.47
Intramucosal cancer	89 (54.3%)	164 (48.7%)	
SM superficial cancer	11 (6.7%)	20 (5.9%)	
SM deep cancer	11 (6.7%)	19 (5.6%)	
Others	0 (0.0%)	2 (0.6%)	
Failure of retrieval	1 (0.6%)	1 (0.3%)	

### Short-term outcomes of colorectal ESD

A comparison of short-term outcomes following ESD in each group is summarized in Table 2. The mean procedure times were 117 min (range 20–450) in the elderly group and 112 min (range 10–525) in the non-elderly group. There was no significant difference in procedure time. The en bloc resection rates were 96.3% in the elderly group and 93.4% in the non-elderly group. The non-curative resection rate in the elderly group was 7.4%, while the rate in the non-elderly group was 6.8%. There were no significant differences in the en bloc resection rates, non-curative resection rates, and hospital stay lengths between the two groups. Intraoperative perforation and postoperative bleeding rates in the elderly group were 6.1 and 3.0%, respectively, and corresponding values in the non-elderly group were 5.9 and 0.9%. There was one case of delayed perforation in the elderly group (0.6%). The elderly group showed a slightly higher postoperative bleeding rate; however, there was no statistically significant difference. During the study period, four cases with severe complications required emergency surgery. There were no significant differences in the rates of overall complications or emergency surgeries between groups. Of the patients who underwent non-curative resections, five in the elderly group and eight in the non-elderly group were carefully followed without additional treatment. The proportions of patients who required additional surgery were not significantly different between the two groups (Table 3).

**Table 2:** Short-term outcomes of colorectal ESD

	Elderly patients	Non-elderly patients	*p* value
Procedure time (min), mean (range)	117 (20–450)	112 (10–525)	0.12
En bloc resection rate	96.3%	93.4%	0.18
Non-curative resection rate	7.4%	6.8%	0.82
Hospital stay (day), mean (range)	7.5 (5–32)	7.8 (4–27)	0.37
Complication rate (overall)	9.8% (16/164)	6.8% (23/337)	0.25
Intraoperative perforation	6.1% (10/164)	5.9% (20/337)	0.94
Postoperative bleeding	3.0% (5/164)	0.9% (3/337)	0.07
Delayed perforation	0.6% (1/164)	0.0% (0/337)	0.15
Emergency surgery	1.2% (2/164)	0.5% (2/337)	0.46
Cause for emergency surgery (*n*)			
Intraoperative perforation	0	1	
Delayed perforation	1	0	
Postoperative bleeding	1	0	
Intraoperative bleeding	0	1

**Table 3:** Management of patients who underwent non-curative resection

	Elderly patients	Non-elderly patients	*p* value
Additional surgery (*n*)	7/12	15/23	0.69
Residual lesions (*n*)	4/7	3/15	
Lymph node metastasis (*n*)	1/7	4/15	
Distant metastasis (*n*)	0/12	0/23	
Careful follow-up (*n*)	5/12	8/23	

### Long-term outcomes of endoscopic submucosal resection

**Fig. 1:**
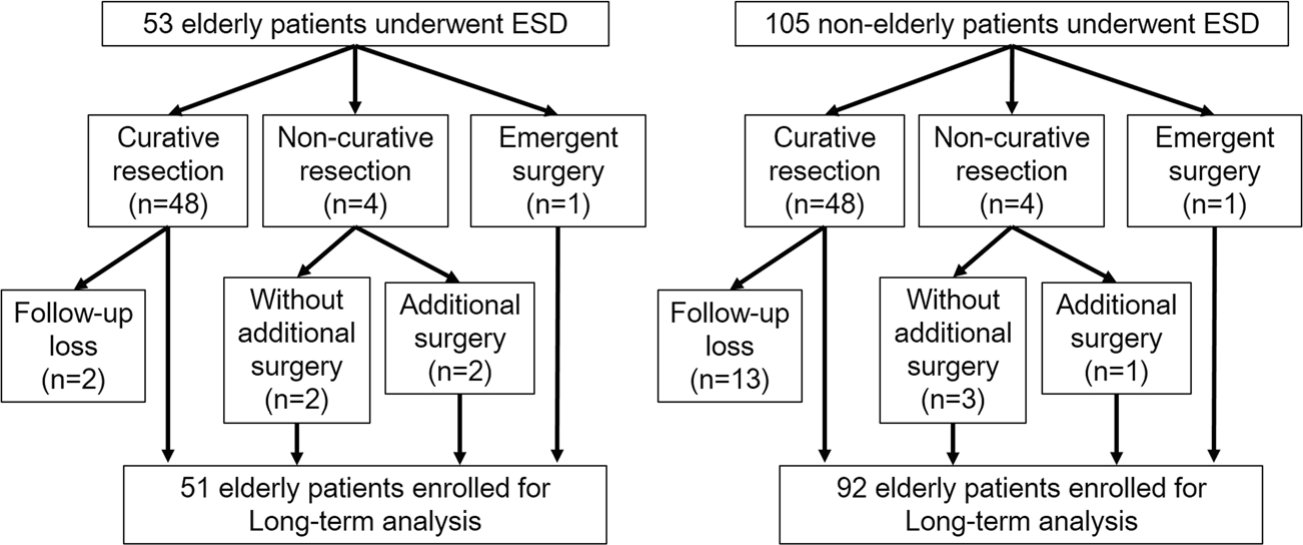
Inclusion of patients in the analysis to evaluate long-term outcomes after colorectal endoscopic submucosal resection (ESD)

Among all patients, 51/53 (96.2%) in the elderly group and 92/105 (87.6%) in the non-elderly group were enrolled in long-term analysis after exclusion of follow-up losses (Fig. 1). There was no significant difference in follow-up rates (*p* = 0.08). Median follow-up duration of the elderly and non-elderly groups was 70.6 and 75.4 months, respectively. Local recurrence was found in one case (2.0%) in the elderly group in which a piecemeal resection was performed.

### Five-year disease-specific survival and overall survival

**Fig. 2:**
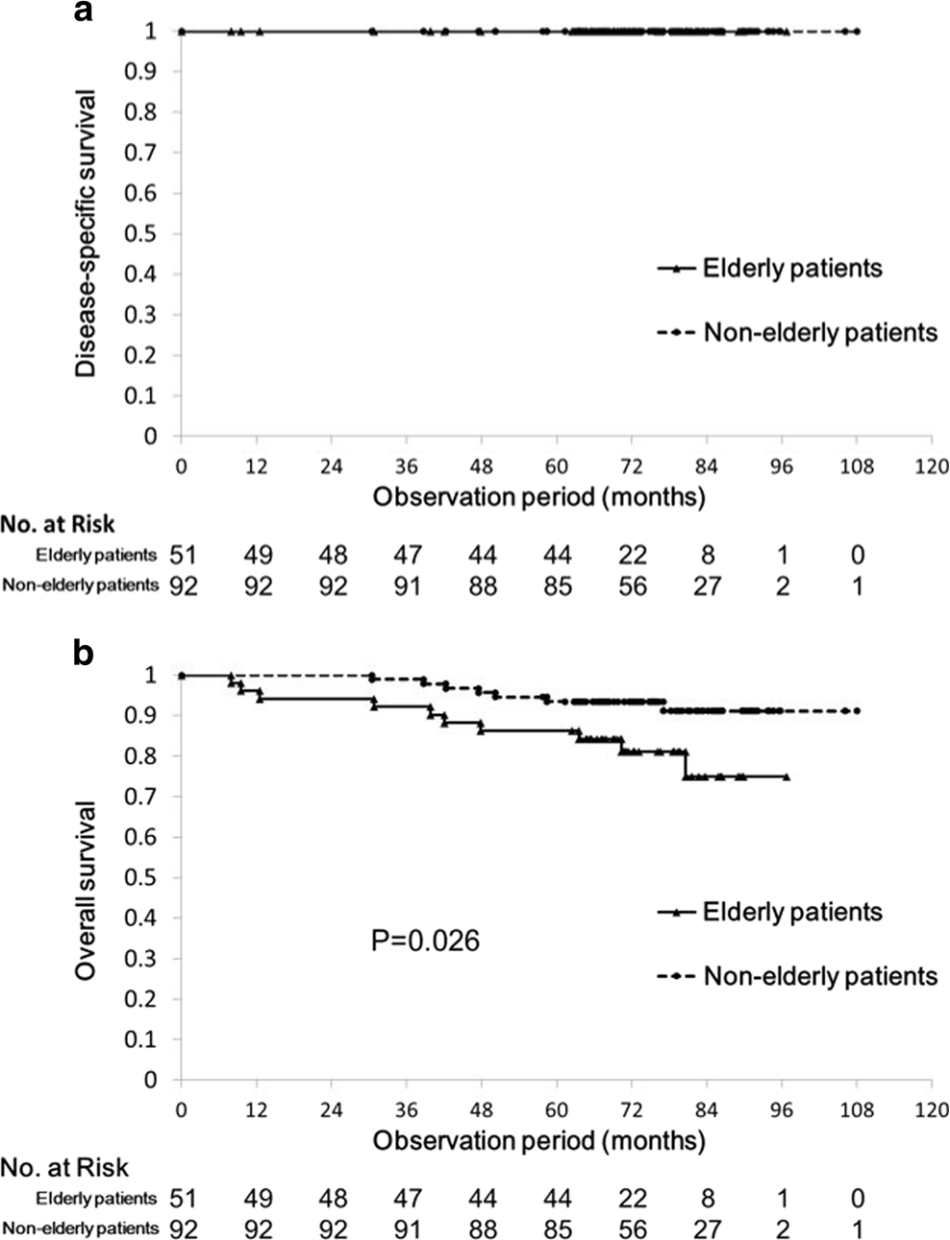
**a** Estimated disease-specific survival curves for the elderly and non-elderly groups. **b** Estimated overall survival curves for the elderly and non-elderly groups. The *solid line* indicates the survival curve of the elderly group, whereas the *dotted line* shows that of the non-elderly group

No disease-specific deaths were noted during the follow-up period. All-cause death was reported in nine patients in the elderly group (17.6%) and nine patients in the non-elderly group (9.8%). The causes of death are summarized in Table 4. Among the causes of death, several types of neoplasms (e.g., lung, liver, stomach, breast, and brain) occurred in both groups. In the elderly group, three patients (33.3%) died of old age. By contrast, three patients (33.3%) died from accidents in the non-elderly group. Figure 2 illustrates the disease-specific and overall survival curves for both groups. The 5-year overall survival rates were 86.3% in the elderly group and 93.5% in the non-elderly group (*p* = 0.026).

**Table 4:** Cause of death during long-term follow-up

Cause of death	Elderly patients (*n* = 9/51)	Non-elderly patients (*n* = 9/92)
Lung cancer	1	1
Liver cancer	2	
Gastric cancer	1	1
Breast cancer		1
Brain tumor		1
Heart attack		1
Renal insufficiency	1	
Pneumonia	1	
Died of old age	3	
Accident	1	3

## Discussion

This study compared short-term and long-term outcomes of colorectal ESD in elderly and non-elderly patients. Safe and effective short-term outcomes of colorectal ESD have been previously reported by several investigators [5–8]. However, few studies have addressed age-specific differences in short-term outcomes [9, 10]. Perforation is one of the complications that could cause a severe adverse event. Several studies have reported that tumor size, morphology, location, and procedure time were important factors associated with intraoperative perforation [16–18]. Regarding patient and lesion characteristics in this study, there were no differences between the two groups except for age. This meant that neither of the two groups had specific risk factors for intraoperative perforation. Intraoperative perforation rates in the elderly and non-elderly groups in our study were 6.1 and 5.9%, respectively. A multicenter questionnaire survey in Japan by Taku et al. [19] and a multicenter study by Tanaka et al. [20] reported that the overall perforation rate of colorectal ESD was 4.8 and 14%, respectively, and these values were comparable to those in our study. Moreover, there was no significant difference in the perforation rates of the two groups. Delayed perforation is a rarer complication than intraoperative perforation that requires emergency surgery [20]. In this study, one case of delayed perforation occurred in the elderly group (2.0%). The risk factors for delayed perforation remain unclear because of the small number of cases published, and therefore further investigation into these risk factors is required.

Bleeding is a common complication of ESD. Most bleeding was generally controllable by using hemostatic devices. However, in this study, there was one case of uncontrollable intraoperative bleeding and one case of postoperative bleeding which required emergent surgeries. Predictive factors for these complications should be clarified by further investigation to ensure safe ESD procedures.

The mean lengths of hospital stay of patients in the elderly and non-elderly groups were 7.5 and 7.8 days, respectively. In our hospital, all patients were admitted on the day before ESD, so the mean hospital stays after ESD in each group were approximately 5 days. Other studies of colorectal ESD reported similar hospital stays [9, 10]. Duration of hospital stay may be a surrogate indicator of postoperative morbidity. There was no significant difference in the length of hospital stays between the two groups; thus, ESD is a well-tolerated procedure for both elderly and non-elderly patients.

Among the patients who underwent non-curative resections, 7/12 (58%) in the elderly group and 15/23 (65%) in the non-elderly group underwent additional surgeries. A previous study reported that the ratio of patients who underwent additional surgeries was lower in elderly patients than younger patients; the reasons for avoiding additional surgery included the low mortality rates for surgery and requests of patients and families [10]. In the current study, the ratio of additional surgeries in the elderly group was slightly lower than in the non-elderly group, but this difference was not statistically significant. According to recent reports, laparoscopic surgery for colorectal cancer shows favorable short-term outcomes in elderly patients as well as in younger patients [21–24]. Recently, most of the additional surgeries have been performed through a laparoscope in our hospital. Therefore, the ratio of elderly patients who undergo additional surgery may be increasing.

Few previous studies have reported the long-term outcomes of patients who underwent colorectal ESD [25, 26]. Niimi et al. [25] reported that the 3-year disease-specific and overall survival rates were 100 and 97.1%, respectively, and the corresponding 5-year values were 100 and 95.3%, respectively. Cong et al. [26] reported similar long-term outcomes of patients with laterally spreading tumors. The median follow-up duration in previous reports was approximately 3 years, whereas patients in our study were followed up for a longer period of time. In addition, this study showed favorable disease-specific survival rates that were comparable to those cited in previous reports. This means that our data provide further support for the benign prognosis of the colorectal lesions resected by ESD. On the other hand, the current study is the first to report age-specific long-term outcomes after colorectal ESD. The non-elderly group showed better overall survival than the elderly group, as expected. In terms of causes of death, there were 4/51 (7.8%) and 4/92 (4.3%) cases with several types of neoplasms in the elderly and non-elderly groups, respectively (*p* = 0.38). Considering the benign prognosis of colorectal lesions resected by ESD, preoperative screening for comorbidities (especially for neoplasia) is important to achieve a better overall survival.

This study has several limitations worth noting. First, this was a single-center retrospective study. Second, few patients developed complications that required an emergent surgery. To accurately determine the risk of severe ESD-associated complications in elderly and non-elderly patients, a further large prospective and multicenter study is required. Third, we did not compare differences in outcomes between endoscopic mucosal resection (EMR) and ESD. According to a systematic review by De Ceglie et al. [27], compared to EMR, ESD showed a higher en bloc resection rate and lower recurrence rate, as well as a higher perforation rate (4.8% in ESD and 0.9% in EMR). Additional disadvantages of ESD include the fact that it is a time-consuming procedure that requires a high level of skill. Therefore, for endoscopists who are not sufficiently familiar with ESD, piecemeal EMR for a large lesion is a reasonable alternative option. At present, there is an ongoing multicenter randomized clinical trial being conducted by Backs et al. [28], the MATILDA trial, which is evaluating the clinical efficacy and cost effectiveness of ESD and EMR.

## Conclusions

Among the patients who underwent colorectal ESD in this study, there were no significant differences in clinical and histopathological characteristics between the elderly and non-elderly groups. ESD showed favorable short-term outcomes and disease-specific survival in both groups. However, the elderly group showed a lower overall survival rate. Considering the higher incidence of comorbidities in elderly patients, preoperative screening is essential for better long-term outcomes.
